# Individual and Community Factors Associated with Household Insecticide-Treated Bednet Usage in the Sunyani West District of Ghana Two Years after Mass Distribution

**DOI:** 10.1155/2020/7054383

**Published:** 2020-09-24

**Authors:** Emmanuel Angmorteh Mensah, Francis Anto

**Affiliations:** ^1^Department of Immunization, Vaccines and Biologicals, World Health Organization Country Office, Kampala, Uganda; ^2^School of Public Health, University of Ghana, Legon, Ghana

## Abstract

**Purpose:**

In the year 2015, the Ghana Health Service launched a free mass insecticide-treated net (ITN) distribution campaign in the Sunyani West district of Ghana with the aim of improving household ownership to increase usage. This study determined the level of ownership and usage of ITNs and associated factors among households in the Sunyani West district two years after the mass distribution campaign.

**Methods:**

Study participants were identified using the systematic approach in all five subdistricts of the Sunyani West district and interviewed, and data were collected on household ITN ownership. Data were also collected on the source of the ITN and whether the respondent slept under an ITN the previous night. Data on individual and community factors associated with ITN ownership and usage were also collected. Pearson chi-square tests and logistic regression were performed to determine factors significantly associated with ITN ownership and usage.

**Results:**

The level of ITN ownership was 78.93% and usage was 55.93%. Most of the participants (73.62%) received their nets during the 2015 mass distribution campaign, 39 (11.96%) received their ITNs during antenatal care visits, whilst 27 (8.28%) bought the nets from the store. People who experience irritation (*χ*^2^ = 23.32; *p* < 0.001) and respondents who did not perceive themselves as likely to be beaten by mosquitoes or get malaria (*χ*^2^ = 26.61; *p* < 0.001) were less likely to use ITNs. Respondents who used other malaria/mosquito bite prevention methods were also less likely to use the ITNs (*χ*^2^ = 206.26; *p* = 0.001), but individuals who received free nets were likely to use them.

**Conclusion:**

ITN ownership was high, but usage was low and far below the national target. Intense health education emphasizing the fact that everybody is susceptible to malaria may help improve usage.

## 1. Introduction

Malaria is a tropical disease of public health importance. It is caused by *Plasmodium* parasites and transmitted through the bites of infected female *Anopheles* mosquitoes. An estimated 219 million (95% confidence interval (CI): 203–262 million) cases of malaria occurred globally in the year 2017. Most of these cases were in the WHO African Region (200 million or 92%) followed by the WHO Southeast Asia Region with 5% of the cases and the WHO Eastern Mediterranean Region with 2% [1].

The World Health Organization's universal plan for malaria control against the high number of cases recommends that malaria control efforts of its member states should ensure that all people living within the high-risk malaria zones are protected through the provision, use, and timely replacement of long-lasting insecticidal nets or, where appropriate, the application of indoor residual spraying [2]. For this reason, insecticide-treated net (ITN) coverage to fight malaria has been increased tremendously by many malaria-endemic countries including Ghana through mass distribution and continuous distribution of ITNs at hospitals and schools. In spite of all these efforts, not much success has been achieved against the fight of malaria because individuals who receive the nets are not using them. It has also been realized that the programmes lack sufficient follow-up and appropriate assessment of factors affecting the use of ITNs [3].

The use of the ITN is considered one of the most cost-effective preventive interventions against malaria as it has been shown to reduce the burden of the disease by 50% in several malaria-endemic settings [4]. Although several strategies including large-scale deployment of ITNs have been implemented by the Ghana Health Service to fight malaria, the disease remains the main cause of illness in the Sunyani West district [5]. Available data indicate that 46% and 39% of all hospital cases in 2013 and 2014, respectively, were due to malaria.

Data from the Demographic and Health Surveys (DHS) indicate a rise in the household ownership of at least one ITN from 42% in 2008 to 68% in 2014. This level of ownership is however still below the target of 100% household ownership set by the National Malaria Control Programme [6].

With the support from donor partners including USAID, ProMPT Ghana, UNICEF, and UKaid, free distribution of long-lasting insecticide-treated net (LLITN) campaign was launched in the Sunyani West district by the Ghana Health Service in 2015. The mass distribution was to increase the household ownership of nets and replace those that were distributed three years earlier in 2012 with the intent to reduce the incidence of malaria in the population.

During the campaign, social mobilization, household registration, and distribution of coupons were undertaken by community volunteers prior to a point distribution exercise. As part of the social mobilization, the public were educated on care and use of the nets. Households were to give priority in terms of net use to pregnant women and children under five years in situations where nets provided were not enough for the entire household. In order to achieve universal coverage of ITNs, one net was allocated to two people in a household (defined as eating or cooking unit). Households with an odd number of people were rounded up to the next even number for net allocation purposes. Although 68,734 nets were required to cover all the registered people, only 55,920 were available for distribution giving 81.4% net coverage through the mass distribution campaign. This study determined the level of household ownership and utilization of ITNs and associated factors in the Sunyani West district of Ghana, two years after the mass distribution.

## 2. Methods

### 2.1. Study Area

The study was carried out in the Sunyani West district in the Brong-Ahafo region of Ghana. The district has five subdistricts, Bofourkrom, Chiraa, Fiapre, Odumase-Kwatire, and Nsoatre, with 85 communities and a population of about 97,700. The total land area is about 1,659 sq km with an average annual rainfall of 1,187 mm and temperature of 25.6°C. The district shares boundaries with the Wenchi district to the northeast, Tain district to the north, Berekum and Dormaa East to the west, Sunyani Municipality to the southeast, and Tano North and Offinso North districts to the east.

The main occupation of the inhabitants is farming with a few engaged in trading activities. The predominant ethnic group is Bono, with a minority group of Ahafo, Dagaaba, Ga, and Ewes. Reports show that although malaria has been the leading cause of illness in the district, the number of cases seen has decreased from 66,129 in 2014 to 44,628 in 2016. Other leading causes of morbidity in the district are upper respiratory tract infections, skin infections, and diarrhea diseases.

### 2.2. Study Design

This was a cross-sectional study conducted among households selected from five communities, one community from each subdistrict within the Sunyani West district. The communities selected were Adantia, Attakrom, Amanfuso, Dumesua, and Bofourkrom. Participants were enrolled into the study using the systematic approach. Data on ITN ownership and usage were collected from the household heads or their representatives (18 years or older) during the study. A structured questionnaire with questions on ITN ownership, usage, and factors likely to influence usage was used for the data collection. The data collection lasted for six weeks during the months of May and June, 2017.

### 2.3. Sample Size Estimation

A sample size of 418 was estimated using ITN usage among households in the Brong-Ahafo region of 44.95% [7]. The Cochran formula [8], *n* = (Z^2^*pq*)/*d*^2^, where *n* = estimated sample size, *Z* = the *z*-score that corresponds with 95% confidence interval (1.96), *p* = estimated proportion of ITN usage (44.95%) = 0.4495, *q* = estimated proportion of households who do not use the ITN (1 − 0.4495 = 0.5505), and *d* = margin of error set at 5% (0.05), was used. 10% allowance was made for non- and incomplete response.

### 2.4. Sampling

The Sunyani West district has five sub districts, and these served as strata from which one community was randomly selected. After the five communities have been selected from the subdistricts, the predetermined sample size was distributed proportionately per the population of the communities, as registered by the community volunteers in the 2015 mass ITN distribution campaign. In each community, the community household registers developed between February and April were used as the sampling frame. The total number of households in the register was divided by their share of the sample size. Systematic method was then used to select households for the study. The first household was selected using the random number generator to pick a household within the first sampling interval of the systematic selection. In the communities, selected households were identified by the help of a community-based surveillance volunteer or an opinion leader. In situations where the selected household had no one temporary available to be interviewed, a revisit was scheduled. Households that have permanently moved are replaced.

### 2.5. Clustering Effect

Individuals within the same community are likely to be more similar to each other. To minimize this effect, a binary outcome variable was used. Clustering effects are less prominent in binary outcomes [9]. The study focused on individual-level analysis. ITN use and its associated factors were assessed for the individual respondent on the assumption that observed characteristics are independent of others [10, 11]. The Sunyani West district was considered as a unit. Geographical stratification, selection of communities from all five subdistricts using simple random sampling and application of two-stage sampling, was used purposely to ensure representativeness.

### 2.6. Data Collection

Trained research assistants moved from house to house within the selected communities and collected data from the household heads or their representatives onto a pretested structured questionnaire developed specifically for this study. The interviews were conducted in English or Twi/Bono as determined by the study participant. Data on sociodemographic characteristics including area of residence, age, marital status, educational level, occupation, and religion were collected directly from participants through face-to-face interviews. These interviews lasted for about 30–45 minutes, and revisits were scheduled when participants were not met in their homes during the first visit.

Data were also collected on the household ownership of the net, number of nets, source of the net, number of nets hanged, reasons for not owning a net, and whether one slept under an ITN the night before the data collection. Other information collected was experiencing burning sensation when one slept under an ITN, perceived susceptibility to malaria, use of alternative malaria/mosquito bite prevention methods, and whether household received a free net during the 2015 mass distribution campaign.

### 2.7. Data Processing and Analysis

The data were entered using Excel version 2013 software, imported to Stata (Stata 14) and cleaned before analysis. Categorical variables were summarized into frequencies and proportions. Continuous variables such as age were summarized into means and ranges and categorized into age groups. Bivariate analysis was done using Pearson chi-square tests to assess significant differences between ITN ownership and usage and categorical variables. Factors with *p* value <0.05 at 95% CI were considered statistically significant.

### 2.8. Quality Control

Five research assistants were trained over a period of five days on how to effectively conduct interviews and also how to handle ethical issues including how to obtain written informed consent. The questionnaire was pretested in Yawhima, a community in Sunyani Municipality. The questionnaire was reviewed after pretesting based on problems identified during the exercise.

### 2.9. Potential Limitations of the Study

The study was carried out in May–June. The period is a major rainy season in Ghana. Mosquito density is usually high, and ITN usage may be higher than usual. The reported ITN usage in this study may be on the higher side.

### 2.10. Ethical Consideration

The study proposal was submitted to the Ghana Health Service Ethical Review Committee for review and approval before commencement of data collection (ethical review number: GHS-ERC: 65/02/17). Written informed consent was obtained from each participant after the purpose of the study had been thoroughly explained to him/her before the interviews were conducted.

## 3. Results

### 3.1. Background Characteristics of Participants and ITN Ownership

A total of 413 household heads aged 18–75 years (mean: 38.99 years and SD ± 12.08 years) participated. The 413 households had a total of 2,359 individuals resident in various households. Out of this number, 556 (23.6%) were children under five years of age. The mean household size was 5.7 (SD: 3.48). From Table 1, 86.20% (367/413) of household respondents were females. About 71.19% (294/413) were married, 22.03% were single, 3.63% divorced, and 3.15% were widows. Most of the respondents, 41.89% (173/413) had primary and JSS education, and 61.50% (254/413) were self-employed.

### 3.2. Household Ownership, Source, and Usage of the ITN

Insecticide-treated net ownership was high in the Sunyani West district with 78.93% (326/413) of households with at least one ITN. A total of 817 nets were in possession of study participants out of which 606 (74.17%) were hanged at the time of data collection. Net density (average nets per person) was 0.35 in households. Out of the 326 participants who had ITNs, 240 (73.62%) got theirs during the 2015 mass distribution campaign, 39 (11.96%) received their ITNs during antenatal care visits, whilst 27 (8.28%) bought the nets from the store. The rest of the participants acquired their nets from child welfare clinics (CWC) and schools. For those who did not have nets, 40.23% said that they prefer to use other methods, 35.63% did not have enough money to buy, 19.54% were of the view that mosquito nets are not easily available to buy.

In all, 182/413 (44.07%) of the respondents surveyed did not sleep under an ITN the night before the survey, with as many as 97/326 (29.75%) of those with ITNs were also not using them the previous night, and 92/319 (28.84%) of the hanged ITNs were also not used. Most of the participants (65.37%) who slept under ITNs the previous night indicted that they used them in order to prevent mosquito nuisance and bites. Net use was significantly associated with sex, occupation of the respondent, and religion. Marital status and education have no significant association with mosquito net usage (Table 1).

**Figure 1:**
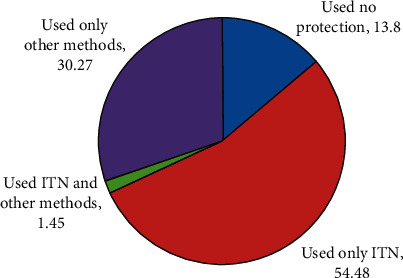
A pie chart showing methods used as protection against malaria among household respondents in the Sunyani West district, June 2017.

The main reason (40.23%, 45/87) for not owning an ITN was the preference for other methods of malaria or mosquito bite prevention including insecticide spray, mosquito coil, and mosquito repellants. Some of the household heads (30.27%) also indicated that they use only other methods in the prevention of malaria (Figure 1). Reasons given for using other malaria/mosquito bite prevention methods were other methods were easy to apply, were cheap to buy, and were easily available.

### 3.3. Factors Associated with ITN Usage

People who experience irritation when they sleep under ITNs were less likely to use them compared to those who do not (*χ*^2^ = 23.32; *p* < 0.001). Similarly, respondents who did not perceive themselves as likely to be beaten by mosquitoes or get malaria were less likely to sleep under the ITNs compared to those who felt they were susceptible (*χ*^2^ = 26.61; *p* < 0.001). Respondents who used other malaria/mosquito bite prevention methods were also less likely to use the ITNs compared to those who did not use such methods (*χ*^2^ = 206.26; *p* = 0.001), but individuals who received free nets were likely to use them (Table 2). In the unadjusted models, hypersensitivity/burning sensation (OR = 0.35, 95% CI (0.222–0.536)), perceived insusceptibility to mosquito bite/malaria (OR = 0.31, 95% CI (0.99–0.491)), and use of other methods (OR = 0.012, 95% CI (0.005–0.029)) reduced the odds that an individual will use the ITN significantly. Receiving free nets through the mass distribution increases ITN use by 4.47 times (95% CI, 2.876–6.944). However, when hypersensitivity/burning sensation, perceived insusceptibility to mosquito bite/malaria, use of other methods, and receipt of free nets were adjusted, only receipt of free nets (AOR = 4.83, 95% CI (2.585–9.035)) and use of other methods (AOR = 0.009, 95% CI (0.003–0.026)) were statistically significant (Table 3).

From Tables 2 and 3, media exposure to messages on ITN usage and malaria in the past six months significantly increased the use of ITNs (*p* = 0.002), health access (respondents residing in communities with health facility) (*p* = 0.017), and mass distribution of ITNs in an area (*p* < 0.001) were associated with ITN use. A simple logistic regression showed that mass distribution promotes ITN use significantly (OR = 3.10, 95% CI (1.603–5.981)), likewise media exposure (OR = 1.97, 95% CI (1.267–3.063)). However, access to healthcare reduced the odds of using a net, and also, individuals in rural communities were more likely to sleep under ITNs, although the difference was not statistically significant. Individuals from rural communities are more likely to use ITNs as compared to peri-urban although this assertion is not statistically significant controlling for media exposure to malaria messages, access to healthcare, and mass distribution.

## 4. Discussion

A community-based study on ITN usage among households was carried out in the Sunyani West district of the Brong-Ahafo region of Ghana. Ownership of ITNs was found to be high (78.93%), but usage was low (55.93%) and influenced by factors such as sensitivity to the chemicals used in treating the net, perceived insusceptibility to malaria, and the preference for other malaria or mosquito bite prevention methods such as the use of mosquito spray and coils. The level of ITN usage was far lower than what was found in Ethiopia, where the usage of ITNs was 85.1% [12], but much higher than that of Tanzania with ITN usage of 32.8% [13].

Households with at least one ITN among the study population was high, with 79% of households having nets that could be used while sleeping, similar to the study in Northern Ghana [14]. Despite the ownership being high, the district is yet to attain the NMCP's target of 100% of households owning at least one ITN. Household ownership of ITNs has seen much increase compared to the coverage in the 2014 Ghana Demographic and Health Survey of 44.95% household ownership of at least one net in the Brong Ahafo region. The high ownership of nets in the district may be due to the mass distribution campaign carried out in 2015. In addition to the mass distribution, there are other distribution outlets such as child welfare clinics (CWC) and antenatal care (ANC). Several studies have reported such high ITN ownership in other malaria-endemic communities. A study by Ernst et al. [15] in Mozambique showed a similar high ownership of nets (78%). Kateera et al. [16] studied long-lasting insecticidal net sources, ownership, and use in the context of universal coverage of households in eastern Rwanda and reported an overall ownership of at least one net to be 92%. ITN ownership was found to be equally high in Benin (84.8%) through a similar study by Tokponnon et al. [17]. From the current study, net density (average nets per person) was 0.35 in the sampled households. This is below the target of the National Malaria Control Programme of achieving universal coverage (one net per two people). The World Health Organization defines universal coverage as one LLIN for every two people at the risk of malaria [18]. This means that net density should be at least 0.5. There is therefore a significant difference in the existing net density and the expected density. The current findings support the report of the global fund that evaluated the malaria programmes in Ghana from 2003 to 2010 and revealed that there was no significant change in the malaria disease burden over the ten-year evaluation period. The lack of improvement was attributed to interventions such as ITNs not reaching enough of the population [19]. A similar situation was observed in Central Uganda, where the percentage of households with LLINs per two people was found to be low (51%) [20], and in Nigeria, a study identified an intrahousehold possession gap (not enough for every family member) as 66% [21].

A statistically significant association was also found between ITN use and receipt of free nets and the use of alternative malaria prevention methods. Also, reasons given in the current study for nonownership and usage of ITNs were similar to those [22] given in a similar study in the Abia state of Nigeria which included “having door and window netting,” “use of other malaria prevention methods,” and “fear of side effects.” Individuals who received free nets through the mass distribution were 4.47 times more likely to use the nets compared to those who did not receive any nets.

With the community factors, media exposure to malaria messages, access to healthcare, and mass distribution influenced ITN use. Access to healthcare would have been predicted to increase net use just as it increases health care utilization, but on the contrary, this reduced net usage in communities that have health facilities. It could be that the presence of health facility indirectly assures community members of availability of treatment/care for malaria, so few efforts are put in to prevention. From the current study, media exposure promoted net usage. The results on the valuable role of media messages are in conformity with the results of Owusu Adjah and Panayiotou in 2014 [23]. Explanation for mass distribution increasing ITN use may be because of increased household ownership and availability of the net. Also, mass distribution campaigns are associated with education on ITN use which also promotes ITN use. A study in Benin showed a positive impact of mosquito net distribution on household ownership and usage [17].

About 30% of the study population used only other malaria prevention methods to protect themselves against malaria. This finding is similar to the finding (44.5%) of Ozims and Eberendu [24] in Imo State, Nigeria. The methods mostly used from the study included insecticide spray (45%) and burning of mosquito coils (41%). Only 5% used mosquito repellents. This practice was higher in peri-urban as compared to rural communities. Some individuals use alternative methods for the lack of mosquito nets, but other reasons given were that the alternative methods used were easy to apply as compared to ITNs, less costly, and also readily available. The proportion of other malaria prevention methods' users was quite substantial, but the Ghana Health Service and National Malaria Control Programme did not recognize or recommend these methods against malaria prevention. A few studies are already against the use of other methods with reasons that they inflict some economic burden on households with no resultant decrease in the risk of developing malaria [25]. However, more must be done to establish the facts for or against the use of other means.

## 5. Conclusion

ITN ownership has increased by 34% since the 2015 mass distribution exercise, but usage of the nets was found to be low and much less than the target set by the National Malaria Control Programme. The situation is worrying as this study was carried out during the rainy season, a time when mosquito density is usually high and ITN usage is expected to be high. Nonuse of nets was significantly associated with perceived insusceptibility to malaria and feeling of burning sensation when sleeping under ITNs. Aside the poor usage of ITNs, significant proportion of individuals uses other methods of mosquito bite prevention. If malaria remains the leading cause of morbidity in Sunyani West and Ghana as a whole, then the alternative methods being used are ineffective. Efforts must be geared towards studies on the effect and effectiveness of popular alternative methods being used and diversification of malaria control methods to give individuals options while maintaining intense ITN use campaigns emphasizing the fact that everybody is susceptible to malaria.

## Figures and Tables

**Table 1:** Demographic factors associated with ITN use among household respondents in the Sunyani West district, June 2017.

Variable	ITN use		Chi-square	*p* value
	Yes	No		
*Sex*				
Male	43 (75.44%)	14 (25.56%)	10.21	0.001
Female	188 (52.81%)	168 (47.19%)		

*Age*				
18–49	181 (45.48%)	151 (54.52%)	5.06	0.08
50–64	43 (32.81%)	21 (67.19%)		
65+	7 (58.82%)	10 (41.18%)		

*Marital status*				
Married	161 (54.76%)	133 (45.24%)		0.097^*∗*^
Single	53 (58.24%)	38 (41.76%)		
Divorced	6 (40.00%)	9 (60.00%)		
Widowed	11 (84.64%)	2 (15.38%)		

*Education*				
No formal education	57 (63.33%)	33 (36.67%)	3.65	0.301
Primary/JSS	97 (56.07%)	76 (43.93%)		
Secondary/Vocational	48 (53.33%)	42 (46.67%)		
Tertiary	29 (48.33%)	31 (51.67%)		

*Occupation*				
Public	41 (51.90)	38 (48.01%)	12. 23	0.007
Private	40 (76.92%)	12 (23.08%)		
Self-employed	132 (51.97%)	122 (48.03)		
Unemployed	18 (64.29%)	10 (35.71%)		

*Religion*				
Christian	186 (52.99%)	165 (47.01%)		0.001^*∗*^
Moslem	37 (72.55%)	14 (27.42%)		
Traditionalist	8 (100.00%)	0 (0.00%)		
Others	0 (0.00)	3 (100.00)		

^*∗*^Fisher's exact test.

**Table 2:** Individual and community factors associated with ITN use among household respondents in the Sunyani West district, June 2017.

Factors	Household respondents	
	Chi-square	*p* value
*Individual factors*		
Sensitivity/burning sensation	23.32	<0.001
Perceived insusceptibility to mosquito bite/malaria	26.61	<0.001
Receipt of free nets	47.18	<0.001
Use of alternative methods	206.26	<0.001

*Community factors*		
Media exposure to malaria messages	9.10	0.002
Mass distribution	17.84	<0.001
Access to healthcare	5.72	0.017
Community classification	4.17	0.014

**Table 3:** Logistic regression of individual factors and community factors associated with ITN use among household respondents in the Sunyani West district, June 2017.

Factor (ref = no)	Crude odds ratio (OR)	95% confidence interval	Adjusted odds ratio (AOR)	95% confidence interval
*Individual factors*				
Hypersensitivity/burning sensation	0.35	0.222–0.536 (*p* = 0.001)	1.48	0.694–3.161 (*p* = 0.310)
Perceived insusceptibility	0.31	0.199–0.491 (*p* = 0.001)	0.55	0.278–1.084 (*p* = 0.084)
Receipt of free nets	4.47	2.876–6.944 (*p* = 0.001)	4.83	2.585–9.035 (*p* = 0.001)
Use of alternative methods	0.012	0.005–0.029 (*p* = 0.001)	0.009	0.003–0.026 (*p* = 0.001)

*Community factors*				
Media exposure	1.97	1.267–3.063 (*p* = 0.003)	2.18	1.320–3.588 (*p* = 0.002)
Access to healthcare	0.52	0.299–0.893 (*p* = 0.018)	0.33	0.180–0.622 (*p* = 0.001)
Mass distribution	3.10	0.299–0.893 (*p* = 0.001)	2.9	1.438–5.923 (*p* = 0.003)
Community classification	1.53	1.016–2.308 (*p* = 0.042)	1.26	0.821–1.947 (*p* = 0.286)

## Data Availability

The data used to support the findings of this study are available from the corresponding author upon request.
